# Cell-specific exosomes in sepsis-associated ARDS: from immunometabolic reprogramming to precision medicine

**DOI:** 10.3389/fimmu.2026.1799086

**Published:** 2026-03-27

**Authors:** Yihan Dang, Caifeng Yan, Haiying Rui, Xiaoxi Yan, Miaobo Li, Xiaorong Dong, Li Ma

**Affiliations:** Department of Critical Care Medicine, The Second Hospital & Clinical Medical School, Lanzhou University, Lanzhou, Gansu, China

**Keywords:** sepsis, acute respiratory distress syndrome, exosomes, ferroptosis, cuproptosis, immunometabolism, liquid biopsy

## Abstract

The publication of the 2023 Global Definition of ARDS has further unveiled the clinical heterogeneity of sepsis-induced acute respiratory distress syndrome (ARDS), rendering traditional systemic biomarkers insufficient for precisely characterizing lung-specific pathological changes. Cell-specific exosomes, owing to their high stability and high fidelity to the molecular signatures of their parent cells, have emerged as a highly promising tool for liquid biopsy. This review aims to elucidate how exosomes construct a multidimensional communication network within the compromised alveolar-capillary barrier. Beyond exploring the traditional function of exosomes as inflammatory vectors, we provide an in-depth analysis of the mechanisms by which alveolar epithelial exosomes propagate ferroptosis and mitochondrial damage in a wave-like manner, and how macrophage exosomes drive immunometabolic reprogramming via glycolysis and histone lactylation to sustain the inflammatory state. Furthermore, we elaborate on the central role of endothelial exosomes in vascular leakage and immunothrombosis, proposing a novel hypothesis that they may serve as mediators propagating cuproptosis within the vascular bed. Finally, by integrating advances in single-cell omics and analyzing technical barriers such as isolation specificity and timeliness, we propose a precision medicine framework based on exosomal molecular fingerprints. This strategy aims to utilize exosomes for ARDS subphenotyping, thereby promoting a paradigm shift in clinical practice from syndrome management to mechanism-driven theranostics.

## 1 Introduction

Sepsis-induced acute respiratory distress syndrome (ARDS) remains one of the clinical syndromes with the highest mortality rates in critical care medicine. Its pathophysiological characteristics mainly manifest as diffuse alveolar damage, increased pulmonary microvascular permeability, and refractory hypoxemia (1). For a long time, clinical diagnosis has primarily relied on the 2012 Berlin Definition, which grades severity based on the oxygenation index and positive end-expiratory pressure levels (2). However, the release of the 2023 Global New Definition of ARDS marks a turning point in this field (3). This new standard expands the diagnostic scope to include non-intubated patients receiving high-flow nasal oxygen (HFNO) and permits the use of pulse oximetry as a surrogate for arterial blood gas analysis in resource-limited settings, thereby significantly improving diagnostic sensitivity and advancing the identification window.

Despite the fact that the implementation of the new definition aids in early intervention, the ensuing “clinical heterogeneity paradox” presents immense challenges for precision treatment. With the inclusion of non-intubated patient populations and the diversification of hypoxia assessment methods, confirmed patients exhibit unprecedented variability in pathophysiological basis, disease severity, and therapeutic responsiveness (4). In this context, traditional systemic inflammatory biomarkers such as C-reactive protein (CRP) or interleukin-6 (IL-6), while effective for infection monitoring, lack organ specificity against the backdrop of complex systemic inflammatory responses. They struggle to precisely identify specific pulmonary pathological events, such as alveolar epithelial injury, endothelial barrier disruption, or microcirculatory dysfunction (5).

To break through this bottleneck, exosomes are emerging as key carriers for resolving ARDS heterogeneity due to their unique biological properties. Although the latest guidelines released by the International Society for Extracellular Vesicles (ISEV) recommend prioritizing the term “small extracellular vesicles” based on physical characteristics (6), considering the widespread usage of the term “exosome” in the field of sepsis research, this article will uniformly use “exosomes” to refer to these membrane-bound vesicles with diameters ranging from 30 to 150 nanometers and biological activity (7). Unlike unstable free proteins, the lipid bilayer structure of exosomes effectively protects their encapsulated contents—including proteins, nucleic acids, and lipids—from degradation, endowing them with extremely high stability in the circulatory system (8). More critically, the exosomal membrane surface retains specific antigens derived from parent cells, such as the Receptor for Advanced Glycation End-products (RAGE) from alveolar epithelium or Cluster of Differentiation 31 (CD31) from endothelial cells, enabling them to serve as liquid biopsy tools carrying cell-of-origin information to reflect microenvironmental changes in lung tissue in real-time (9).

**Figure 1:**
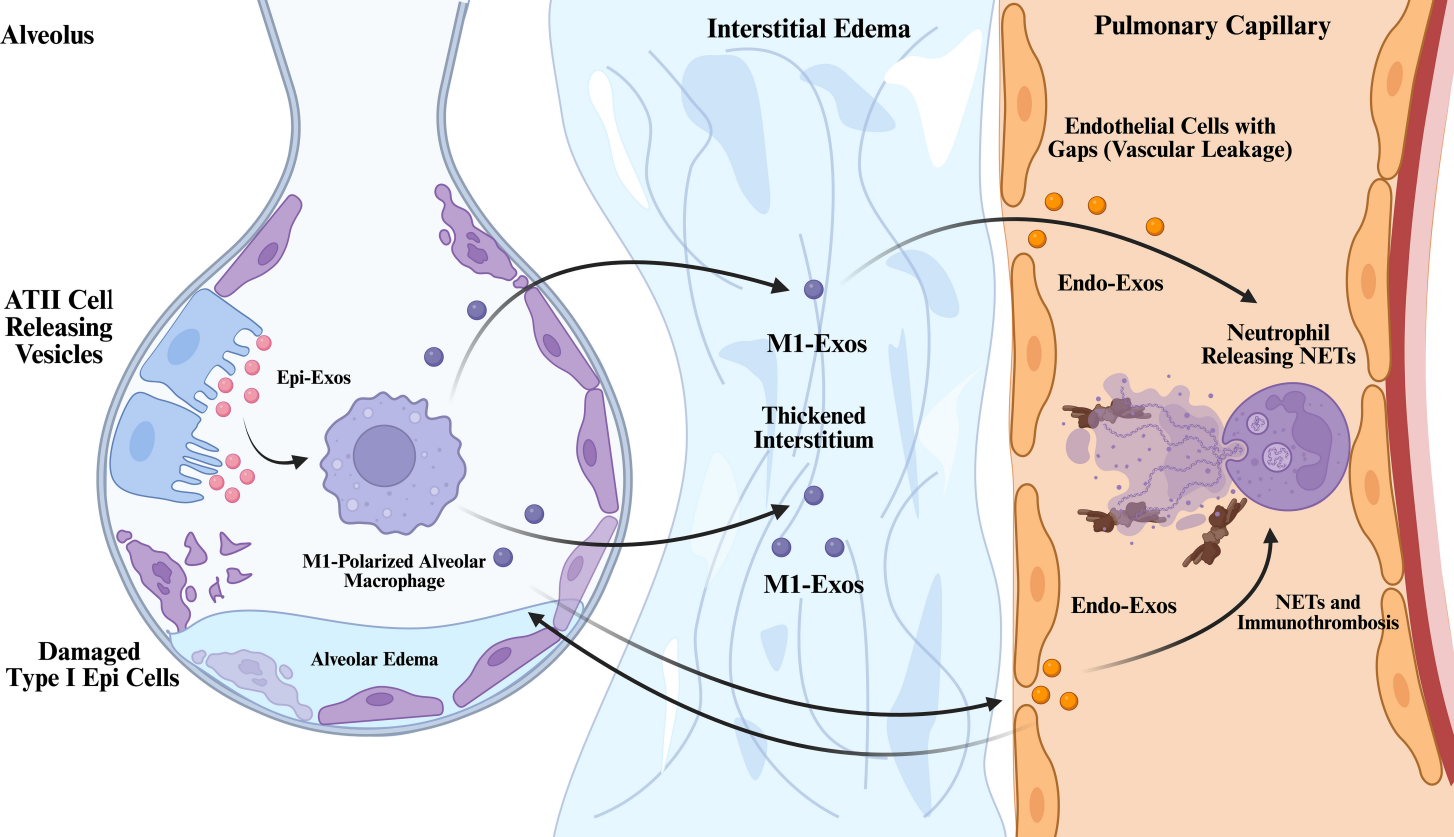
The multidimensional exosomal communication network across the alveolar-capillary barrier in sepsis-associated ARDS.

This review aims to transcend the traditional framework of inflammation description and, combining the latest research progress, systematically elucidate the multidimensional regulatory network of cell-specific exosomes in sepsis-induced ARDS (**Figure 1**). We will also delve into the cascade propagation mechanisms of novel forms of cell death, such as ferroptosis and cuproptosis (10), analyze the central role of immunometabolic reprogramming and lactylation modification in intercellular communication (11), and integrate single-cell sequencing and multi-omics technologies to propose new strategies for ARDS precision subtyping and theranostics based on exosomal atlases, providing a solid theoretical basis for clinical translation in this field.

This figure depicts the cascade propagation process of injury signals mediated by exosomes. Under sepsis stress, damaged type II alveolar epithelial cells release exosomes (Epi-Exos, pink), inducing alveolar macrophages to polarize toward the pro-inflammatory M1 phenotype (12). Activated M1 macrophages release exosomes (M1-Exos, purple), which traverse the thickened pulmonary interstitium to transmit inflammatory signals from the air phase to the blood phase (13–15). Damaged endothelial cells release exosomes (Endo-Exos, orange), which synergize with the M1-Exos crossing the barrier to induce neutrophils to release neutrophil extracellular traps (NETs) and promote immunothrombosis (16, 17); additionally, some endothelial exosomes diffuse retrogradely through the damaged barrier. This cross-cellular bidirectional communication constructs a malignant positive feedback loop that sustains inflammation, ultimately leading to vascular leakage and barrier disintegration.

## 2 Literature search strategy and selection criteria

To systematically construct the multidimensional communication network of exosomes in ARDS and minimize the selection bias inherent in traditional narrative reviews, a comprehensive literature search was conducted. Electronic databases, including PubMed, Web of Science, and Embase, were meticulously queried for articles published from database inception to February 2026.

The search strategy integrated Medical Subject Headings (MeSH) and free-text keywords to capture relevant studies comprehensively. The primary search framework employed the following Boolean logic: (“Sepsis” OR “Septic Shock”) AND (“Acute Respiratory Distress Syndrome” OR “ARDS” OR “Acute Lung Injury”) AND (“Exosomes” OR “Extracellular Vesicles” OR “Small Extracellular Vesicles”). Furthermore, the reference lists of key retrieved articles were manually screened to identify additional pertinent evidence.

To ensure the academic rigor and translational relevance of the synthesized evidence, stringent inclusion and exclusion criteria were established. Inclusion criteria comprised: (1) peer-reviewed original research articles and high-quality comprehensive reviews; (2) studies explicitly exploring the pathophysiological roles, intercellular communication mechanisms, or liquid biopsy potential of cell-specific exosomes in sepsis or ARDS models (encompassing both *in vivo* and *in vitro* paradigms); and (3) articles published in the English language. Conversely, the exclusion criteria were: (1) non-English publications; (2) conference abstracts, editorials, case reports, or unpublished preprints lacking rigorous peer review; and (3) studies focusing exclusively on other extracellular vesicle subtypes (e.g., apoptotic bodies) without a definitive mechanistic link to exosomes in the specified pathological context.

## 3 Biogenesis and cargo reprogramming of exosomes in the sepsis environment

In the complex pathophysiological network of sepsis, the release of exosomes is no longer merely a basal metabolic activity of cells but transforms into a precisely regulated mechanism of intercellular stress communication (18). This shift from homeostasis maintenance to pathological adaptation involves alterations in the kinetics of multivesicular body (MVB) generation and the specific remodeling of cargo sorting, constituting the molecular basis of the lung injury cascade (19).

### 3.1 Biogenesis dynamics driven by hypoxia and endoplasmic reticulum stress

Sepsis-induced microcirculatory dysfunction plunges lung tissue into a state of severe hypoxia, and this environmental stress directly reshapes the secretion dynamics of exosomes. Hypoxia-inducible factor-1α (HIF-1α), acting as a key transcriptional regulator, exerts a dual role in the hypoxic environment of sepsis. It not only reprograms cellular energy metabolism pathways by upregulating glycolytic genes (20) but has also been confirmed to directly activate the transcription of *RAB22A*, thereby driving the formation and release of exosomes (21). These hypoxia-induced exosomes exhibit significant heterogeneity in their composition (22); although enriched with angiogenic factors and metabolic regulatory enzymes aimed at restoring the oxygen supply balance of the local microenvironment, they paradoxically exacerbate the disruption of vascular permeability at pathological concentrations (23).

Concurrently, sepsis-induced endoplasmic reticulum stress (ER stress) and the activation of the unfolded protein response (UPR) trigger cellular quality control mechanisms, causing exosomes to become a special channel for cells to clear toxic loads. Cells specifically sort accumulated unfolded proteins, oxidized mitochondrial DNA (mtDNA), and components of the NLRP3 inflammasome into exosomes for expulsion (24, 25). While this process temporarily alleviates proteotoxic stress in the parent cells, these encapsulated dangerous molecules rapidly transform into high-concentration extracellular signaling sources, transmitting injury signals to neighboring alveolar epithelial and endothelial cells, thereby triggering a more extensive inflammatory cascade (26).

### 3.2 Metabolic reprogramming and epigenetic modification of cargo

With the deepening of research since 2024, the academic community has gradually recognized that the pathological functions of sepsis exosomes far exceed the scope of traditional inflammatory factors; their core pathogenicity stems from the transmission of complex metabolic and epigenetic information. At the metabolic level, exosomes released by immune cells and structural cells have been confirmed to be highly enriched in key glycolytic enzymes such as pyruvate kinase M2 (PKM2) and lactate dehydrogenase (LDH), thereby mediating metabolic remodeling in recipient cells (27). Once these biologically active enzymes are taken up, they can rapidly alter the metabolic flux of the recipient, forcing the cell to shift from oxidative phosphorylation to a pro-inflammatory glycolytic state (the Warburg effect), thus reshaping the immunometabolic landscape of the lung tissue (28).

At the epigenetic level, the sepsis-specific high-lactate microenvironment has triggered a paradigm shift in the field of post-translational modifications. The latest evidence indicates that high concentrations of lactate are not merely metabolic waste but key signaling molecules that induce lactylation modifications on histones and non-histone proteins such as high mobility group box 1 (HMGB1) (11). These exosomes carrying lactylated proteins act as epigenetic messengers; upon entering recipient cells, they can directly regulate chromatin accessibility and the binding activity of transcription factors. This solidifies and amplifies the inflammatory response at the gene transcription level, providing a novel molecular perspective for understanding the refractory nature of sepsis-associated ARDS (29).

## 4 Alveolar epithelial cell-derived exosomes: molecular tracing of barrier disintegration and transmission of novel regulated cell death

Alveolar epithelial cells, composed of tightly inlaid flat type I cells and secretory type II cells, serve as the physical cornerstone of the alveolar-capillary barrier and the core unit for maintaining gas exchange and alveolar microenvironmental homeostasis (30, 31). In the pathological progression of sepsis-induced acute respiratory distress syndrome (ARDS), alveolar epithelial cells are not only the primary victims of the cytokine storm but also active participants driving disease deterioration (32). Recent research perspectives have extended from simple cellular structural damage to intercellular communication, revealing that alveolar epithelial-derived exosomes, while carrying injury information, deliver specific molecular cargoes to propagate novel regulated cell death signals within the alveolar space, thereby triggering cascading barrier disintegration and immune dysregulation (33).

### 4.1 Alveolar epithelial-derived exosomes: from molecular tracing to pathological mechanism decoding

The key to precise diagnosis of sepsis-associated ARDS lies in the specific identification of alveolar epithelial-derived exosomes from the complex pool of circulating vesicles. This relies on establishing a dual molecular recognition strategy based on membrane protein tracing and cargo decoding, aiming to transform exosomes from simple biomarkers into dynamic messengers capable of revealing barrier injury mechanisms.

#### 4.1.1 Tracing cornerstone: localization of cell injury based on membrane surface proteins

Specific proteins embedded in the exosomal membrane constitute molecular fingerprints for tracking cell injury. The Receptor for Advanced Glycation End-products (RAGE), as a specific marker of the basolateral membrane of type I alveolar epithelial cells (34), its enrichment on the exosomal surface directly reflects physical cellular disintegration. Moreover, during the progression of sepsis-induced lung injury, this receptor not only sheds from the surface of damaged cells to form soluble isoforms but its levels have also been confirmed to be independently associated with poor prognosis in patients (35). Meanwhile, the release of Surfactant Protein (SP)-positive exosomes reveals metabolic dysfunction and impaired synthesis in type II cells, clarifying the pathological basis of alveolar collapse and microenvironmental instability (36). Additionally, the downregulation or dysfunction of Aquaporin 5 (AQP5), responsible for water transport, marks the loss of epithelial fluid clearance capacity, predicting the trans-barrier exacerbation of non-cardiogenic pulmonary edema from the interstitium to the alveolar space (33, 37). These three categories of markers collectively construct a multidimensional evaluation system ranging from structural destruction and secretion disorders to functional failure.

#### 4.1.2 Pathological decoding: intraluminal cargo-driven inflammatory cascades and death propagation

After completing tracing via membrane proteins, analyzing the molecular cargoes aberrantly sorted into the exosomal lumen can further reveal the molecular mechanisms underlying injury. Post-transcriptional regulatory reprogramming at the nucleic acid level is a significant feature; pro-inflammatory miRNAs (such as miR-92a-3p) transmitted via exosomes drive macrophage polarization and amplify inflammation (12), while protective miRNAs maintaining barrier homeostasis (such as miR-146a) show characteristic depletion, marking the collapse of endogenous protective mechanisms (38). Furthermore, the aberrant transport of long non-coding RNAs (lncRNAs) such as *Rmrp* or *STIMATE* participates in late-stage pathological processes such as immune paralysis by reprogramming immune cell metabolism and calcium signaling (39, 40). At the protein level, damaged epithelial cells release Tenascin-C-rich exosomes to activate the Toll-like receptor 4 (TLR4) pathway (41); more critically, pyroptosis-executing proteins such as Gasdermin D (GSDMD) and Caspase-1 are also encapsulated within (42, 43), transforming exosomes into death messengers that cascade pyroptotic signals to adjacent tissues. This alteration in cargo not only reflects the current pathological state but also reveals the molecular driving forces behind the cytokine storm and barrier disintegration.

### 4.2 New dimensions of barrier disruption: ferroptosis propagation and mitochondrial metabolic-immune crosstalk

**Figure 2:**
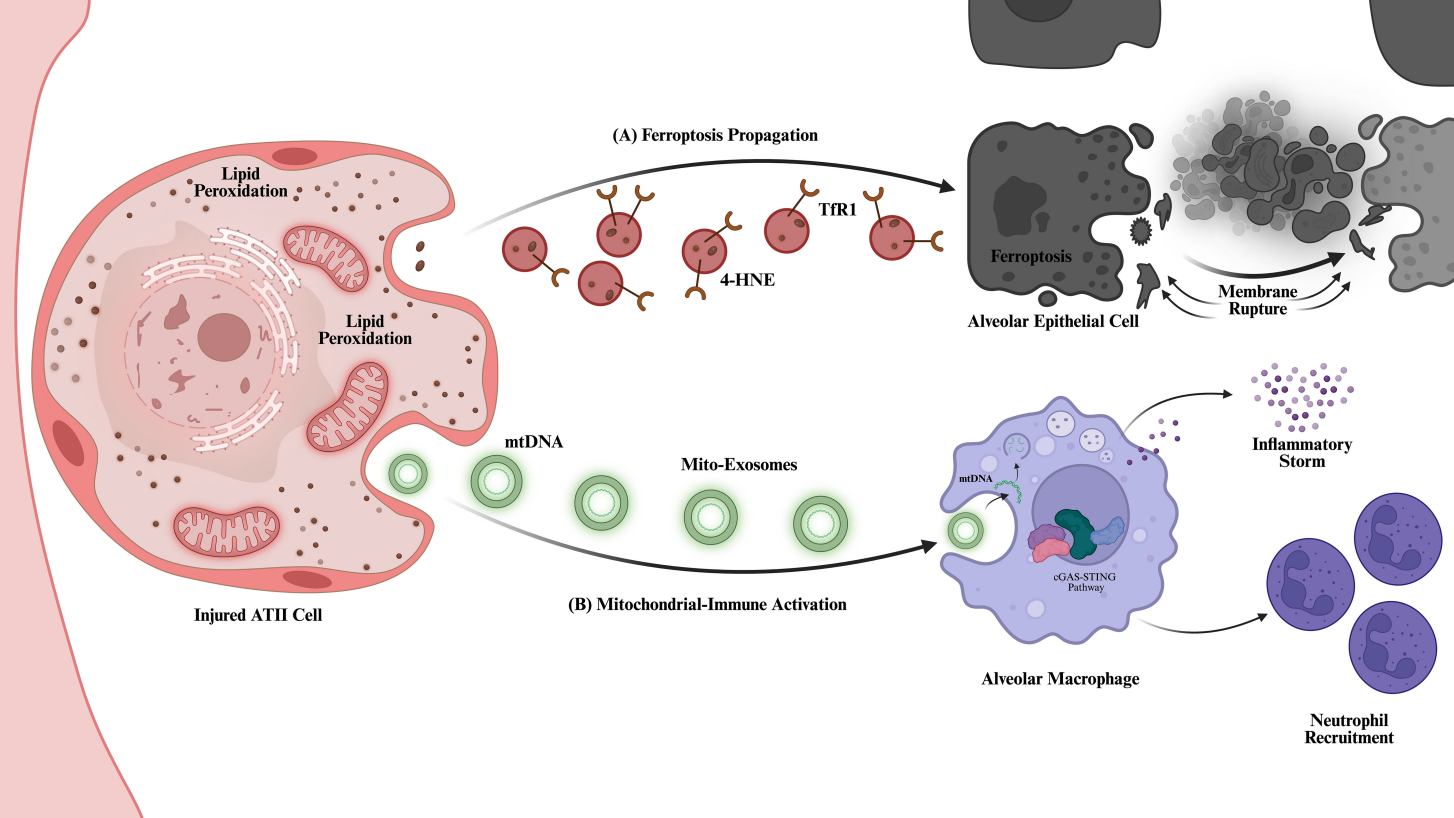
Exosome-mediated ferroptosis propagation and mitochondrial-immune crosstalk mechanisms. **(A)** Paracrine propagation of ferroptosis (upper panel): ATII cells undergoing lipid peroxidation release ferroptosis-related exosomes (red spheres) enriched with Transferrin Receptor 1 (TfR1) and 4-Hydroxynonenal (4-HNE) (46–48). Upon uptake by adjacent healthy alveolar epithelial cells, these vesicles transmit oxidative stress, inducing secondary ferroptosis and membrane rupture, thereby accelerating the disintegration of the alveolar barrier (49). **(B)** Mitochondria-dependent immune activation (lower panel): Damaged ATII cells simultaneously release mitochondrial exosomes (Mito-Exosomes, green rings) encapsulating mitochondrial DNA (mtDNA) (50). After alveolar macrophages engulf these exosomes, the carried mtDNA serves as a danger signal to activate the intracellular cGAS-STING pathway, triggering an inflammatory storm and recruiting neutrophils, thereby amplifying the pulmonary immune-inflammatory response (51, 52).

With the deepening understanding of the pathological mechanisms of sepsis-associated ARDS, research hotspots are gradually shifting from classical apoptosis to more destructive forms of regulated cell death (44). Alveolar epithelial-derived exosomes are not merely products of barrier damage but key intercellular mediators (12), functioning through mechanisms that can drive the cascade diffusion of ferroptosis and bridge metabolic dysfunction with the immune storm (45) (**Figure 2****).**

#### 4.2.1 Ferroptosis-mediated barrier collapse: exosomal relay in the wave of lipid peroxidation

Ferroptosis is an iron-dependent form of cell death driven by lipid peroxidation, and its core status in sepsis-induced lung injury is becoming increasingly prominent (53). Under endotoxin shock, the activity of the key antioxidant enzyme Glutathione Peroxidase 4 (GPX4) in alveolar epithelial cells decreases significantly, leading to the lethal accumulation of lipid peroxides on membrane systems (54). Specific exosomes released by damaged epithelial cells become promoters of this death wave; these vesicles are rich in oxidized lipids such as 4-Hydroxynonenal (4-HNE) modified proteins and are extremely deficient in GPX4 (46, 47). When taken up by adjacent healthy alveolar epithelial cells, they rapidly deplete the recipient cells’ antioxidant reserves and transmit lipid peroxidation stress, thereby inducing secondary ferroptosis responses. This paracrine mechanism mediated by exosomes causes alveolar epithelium to exhibit wave-like collective death, accelerating the total collapse of barrier function (49). Targeting this mechanism, the high expression of Transferrin Receptor 1 (TfR1) on the exosomal membrane and the aberrant loading of ferritin within the lumen constitute specific biomarkers for identifying the occurrence of pulmonary ferroptosis, providing molecular guidance for the precise clinical use of deferoxamine or lipophilic antioxidants (48).

#### 4.2.2 Mitochondrial exosome release: transformation from metabolic dysfunction to innate immune activation

Beyond membrane-level destruction, mitochondrial injury is the initiating factor for irreversible pathological changes in the early stages of sepsis, while the release of exosomes encapsulating mitochondrial components (Mito-Exosomes) transforms this intracellular metabolic catastrophe into strong intercellular immune signals (55). Damaged mitochondria are engulfed into multivesicular bodies via autophagy pathways and subsequently secreted extracellularly as exosomes (50). These special exosomes faithfully carry the oxidized mitochondrial DNA (mtDNA) of the parent cells. When Mito-Exosomes are engulfed by alveolar macrophages, the mtDNA loaded within acts as a potent Damage-Associated Molecular Pattern (DAMP), escaping lysosomal degradation and activating the cyclic GMP-AMP synthase-stimulator of interferon genes (cGAS-STING) pathway or TLR9 signaling axis (51, 52). This process not only triggers an NF-κB-mediated burst of inflammatory factors but also exacerbates neutrophil infiltration, thereby establishing a vicious cycle of epithelial metabolic injury-macrophage activation-neutrophil recruitment. Therefore, detecting circulating exosomes carrying mtDNA is essentially capturing the early signals of pulmonary cellular metabolic collapse and the initiation of the immune storm, providing a highly potential intervention target for intercepting the inflammatory cascade of ARDS.

### 4.3 From mechanism to clinic: new diagnostic and therapeutic strategies based on alveolar epithelial exosomes

Based on the core roles of alveolar epithelial exosomes in barrier disintegration, immune activation, and the propagation of regulated cell death described above, translating them into clinically usable diagnostic and therapeutic tools has become an urgent need for precision medicine (56). This implies not only advancing the diagnostic window but also representing a paradigm shift in therapeutic strategies from systemic anti-inflammation to targeted lung repair.

#### 4.3.1 Liquid biopsy and molecular subtyping of ARDS

Although the traditional Berlin Definition has utility in clinical grading, it ignores the high biological heterogeneity of ARDS (57). Alveolar epithelial-derived exosomes, with their high abundance and membrane structural stability, provide an ideal liquid biopsy medium to bridge this gap (58). The primary direction for clinical translation lies in constructing a molecular subtyping system based on exosomal membrane protein fingerprints. For instance, by quantitatively detecting RAGE and AQP5 levels on the surface of circulating exosomes via flow cytometry, clinicians can not only non-invasively assess the degree of alveolar-capillary barrier permeability impairment but also precisely stratify patients into epithelial injury-dominant or endothelial injury-dominant subgroups (59). This subtyping is crucial for guiding treatment, as epithelial injury-type patients may be more responsive to exogenous surfactant replacement therapies aimed at maintaining alveolar surface tension, while responding poorly to simple fluid restriction strategies (60). Additionally, monitoring the dynamic changes of mtDNA or oxidized lipid cargoes within the exosomal lumen can serve as real-time biomarkers for evaluating the efficacy of anti-inflammatory or antioxidant therapies, with sensitivity significantly superior to lagging changes in oxygenation index, thereby providing molecular-level evidence-based grounds for adjusting personalized treatment plans for critically ill patients (61).

#### 4.3.2 Blocking death propagation and nanodelivery therapy

In the therapeutic dimension, intervention strategies targeting alveolar epithelial exosomes mainly present two directions: blockade and utilization. Given that exosomes carrying ferroptosis signals or inflammasome components are “Trojan horses” exacerbating lung injury, developing specific inhibitors against key enzymes of exosome biogenesis (such as neutral sphingomyelinase 2) (62), or utilizing blood purification technologies (such as affinity adsorption columns) to specifically clear pathogenic vesicles (such as glycosylated exosomes or other injury-associated vesicles) from circulation, holds promise for cutting off the cascade amplification loop of the inflammatory storm and cell death at an early stage (62, 63).

Even more promising is the use of engineered exosomes as targeted delivery vectors for the lung. Research indicates that autologous alveolar epithelial cell-derived exosomes possess a natural tropism for the pulmonary immune microenvironment, enabling precise localization and regulation of target cells such as alveolar macrophages (39). This natural homing ability and low immunogenicity make them demonstrate potential superior to traditional synthetic nanoparticles in pulmonary delivery. Through genetic engineering modifications, they can be loaded with reparative mRNA or anti-inflammatory siRNA (64); particularly when combined with nebulization inhalation technology, these engineered exosomes can directly reach the alveolar space, cross the mucus layer, and be specifically taken up by damaged epithelial cells (65). This mode of administration not only avoids the metabolic inactivation and off-target toxicity of systemic administration but also directly promotes *in situ* regeneration of the damaged barrier and the reconstruction of microenvironmental homeostasis, providing a highly potential nanomedicine solution for conquering the clinical challenge of sepsis-associated ARDS (66).

## 5 Macrophage-derived exosomes: the command center of immunometabolic reprogramming

Alveolar macrophages (AMs) are not only the first line of defense in pulmonary immune defense but also key cells that perceive danger signals and initiate responses during the pathological progression of sepsis-associated ARDS (67). Traditional views have focused primarily on the binary switch of macrophages between the M1 pro-inflammatory phenotype and the M2 anti-inflammatory phenotype, as well as the cytokine storm they secrete. However, recent studies indicate that this process actually involves more complex metabolic reprogramming and epigenetic regulation (68). With breakthroughs in the field of immunometabolism, the latest research landscape reveals a more hidden yet critical mechanism: the functional polarization of AMs is profoundly driven by intracellular metabolic reprogramming and epigenetic mechanisms, and exosomes are the key vectors translating this intracellular metabolic state into intercellular communication signals (69). AMs release specific exosomes driven by their intracellular metabolic status to transmit regulatory signals to the lung microenvironment, thereby reshaping alveolar epithelial integrity and vascular endothelial function (70).

### 5.1 Glycolytic fueling and intercellular transmission of metabolic reprogramming

In the early stages of sepsis-induced ARDS, AMs undergo drastic metabolic reprogramming, rapidly shifting from using oxidative phosphorylation for energy production to aerobic glycolysis—the Warburg effect—to meet the energy and biosynthetic demands for the rapid synthesis of inflammatory mediators (71). This metabolic transformation is not confined to the cell interior but achieves cross-cellular transmission of metabolic information via exosomes.

During the pathological process of acute lung injury, M1-polarized macrophages undergo significant glycolytic reprogramming, leading to the significant upregulation and accumulation of key intracellular glycolytic enzymes such as pyruvate kinase M2 (PKM2) and hexokinase 2 (HK2) (71). Particularly under stimulation by inflammatory signals like lipopolysaccharide (LPS), intracellularly accumulated PKM2 no longer primarily functions as a metabolic enzyme but undergoes non-metabolic nuclear translocation. Inside the nucleus, PKM2 binds with hypoxia-inducible factor-1α (HIF-1α) to form a transcriptional complex, which directly binds to the promoter regions of pro-inflammatory genes like *IL-1β*, initiating their transcriptional expression (72). This mechanism constructs a pathological intracellular positive feedback loop, forcing macrophages to maintain a high-glycolysis, high-inflammation metabolic state (Warburg effect), which in turn leads to the massive release of inflammatory factors and exacerbates lung tissue injury. Furthermore, M1 macrophage-derived exosomes (M1-Exos) carry high abundances of miR-155, which can be taken up by recipient cells. Upon entering cells, miR-155 targets and inhibits key negative regulators such as suppressor of cytokine signaling 1 (SOCS1) (13, 14), relieving its inhibition on the NF-κB pathway and thereby promoting inflammatory signal transduction. Concurrently, miR-155 can synergistically regulate intracellular metabolic targets (such as metabolic genes related to pyruvate dehydrogenase, PDH) (15), driving macrophages toward M1 polarization and amplifying the inflammatory response. This metabolic reprogramming may lead to hindered PDH function, blocking pyruvate entry into the tricarboxylic acid (TCA) cycle, thereby causing intracellular lactate accumulation. The accumulation of lactate not only further solidifies the inflammatory phenotype of the cells but also provides a key substrate basis for subsequent epigenetic modifications such as lactylation (11).

As the disease enters the recovery phase or is induced by an anti-inflammatory microenvironment, alveolar macrophages transform toward the M2 phenotype, and the exosomes they secrete become rich in anti-inflammatory and pro-reparative molecules, becoming key signal carriers for maintaining pulmonary homeostasis and initiating tissue regeneration. M2 macrophage-derived exosomes are rich in miR-223. Upon entering cells, miR-223 on one hand negatively regulates NLRP3 inflammasome activity, inhibiting the maturation and release of IL-1β and IL-18 (73). On the other hand, it reduces the generation of inflammatory factors by blocking the STAT3 signaling pathway (74), thereby attenuating tissue injury at the post-transcriptional level. Beyond anti-inflammatory effects, M2 exosomes directly participate in tissue regeneration processes. Recent studies point out that bone morphogenetic protein receptor 2 (BMPR2) carried by macrophage exosomes can activate the BMPR1B-SMAD1-ID1 signaling axis in alveolar epithelial cells, promoting the proliferation of type II alveolar epithelial cells and their transdifferentiation into type I cells, thereby accelerating the re-epithelialization and functional recovery of the damaged alveolar barrier (75).

### 5.2 Lactate and succinate accumulation: epigenetic codes carried by exosomes

As metabolic products accumulate abnormally in the pathological environment, “metabolic wastes” in the traditional sense, such as lactate and succinate, gradually reveal their messenger functions as non-metabolic signaling molecules. Macrophage-derived exosomes reshape the lung microenvironment at the epigenetic level by carrying these metabolites or proteins modified by them.

Histone lactylation is a novel bridge connecting metabolism and gene expression. The latest research indicates that high concentrations of intracellular lactate during sepsis serve not only as an energy source but also as an epigenetic modifier, leading to lactylation modification at lysine 18 of histone H3 (H3K18la) (76, 77). Although this modification promotes the expression of specific repair genes to a certain extent, it may also abnormally activate pathogenic genes in the late stage of the disease, inducing pathological fibrosis (78). Exosomes carrying lactylated histones (such as H3K18la) or lactylated high mobility group box 1 (HMGB1) constitute novel indicators reflecting the metabolic-epigenetic cross-regulation of the organism after being released into the extracellular space. Given the pivotal role of lactate metabolism in pulmonary inflammation, this suggests the potential clinical value of assessing ARDS severity and prognosis from a metabolic dimension (11, 77).

Concurrently, succinate accumulation caused by the interruption of the TCA cycle triggers widespread protein succinylation. Succinylation of PKM2 promotes its transition from a tetramer to a dimer and its entry into the nucleus, subsequently activating the NLRP3/AIM2 inflammasome and exacerbating macrophage pyroptosis and the release of inflammatory factors (IL-1β, HMGB1) (79, 80). This metabolite-driven post-translational modification endows exosomes with deeper destructive power within the cytokine storm, making them key nodes connecting metabolic disorders with cell death.

### 5.3 From monitoring to remodeling: translational medicine prospects of macrophage exosomes

Given the high plasticity of macrophages during the initiation and resolution phases of ARDS inflammation, transforming their exosomes from simple pathological products into clinical diagnostic and therapeutic tools represents a significant breakthrough direction in the field of critical care immunology. This not only promises to solve the problem of the lack of specificity in traditional inflammatory indicators but also provides natural nanocarriers for achieving precise regulation of immune responses.

#### 5.3.1 Dynamic immune phenotyping and immune window identification

One of the greatest dilemmas currently facing the clinic is the inability to precisely judge whether a patient is in the pro-inflammatory storm phase or the immune paralysis phase, making the timing of immunomodulatory therapy difficult to grasp. Macrophage-derived exosomes carry real-time phenotypic information of parent cells, providing a dynamic liquid biopsy window to solve this problem. By detecting the changes in the ratio of M1 markers to M2 markers in circulating exosomes, clinicians can construct a real-time fingerprint of the patient’s immune status (81). This molecular-level phenotyping is significantly superior to traditional white blood cell counts or C-reactive protein (CRP) because it directly reflects the functional polarization state of intrapulmonary immune cells rather than a simple increase or decrease in number. For example, if sustained high expression of M1 exosomes is detected early in the disease course, it suggests the need for intensified anti-inflammatory intervention; conversely, if M2 exosome release is insufficient in the later stages, it predicts impaired repair initiation and a risk of pulmonary fibrosis, at which point the therapeutic focus should shift to pro-reparative strategies (82). This spatiotemporal resolution capability based on exosomal atlases makes it possible to administer the right immunotherapy at the right time, greatly enhancing the success rate of personalized treatment for ARDS.

#### 5.3.2 Targeted reprogramming and nano-immunotherapy

In terms of therapeutic strategies, utilizing exosomes for *in situ* reprogramming of macrophages has become an emerging direction surpassing traditional anti-inflammatory drugs. Unlike the systemic non-specific suppression of corticosteroids, engineered exosomes can achieve targeted regulation of alveolar macrophages. On one hand, mesenchymal stem cells (MSCs) or M2 macrophage-derived exosomes can be modified using genetic or metabolic engineering technologies to overexpress anti-inflammatory miRNAs, metabolic regulatory enzymes, or surface targeting ligands. These therapeutic vesicles, when injected via the airway or vasculature, can be specifically taken up by pro-inflammatory macrophages, forcibly inducing their conversion to the reparative M2 phenotype through epigenetic or metabolic pathways, thereby actively terminating the inflammatory response and initiating tissue regeneration (83). On the other hand, targeting pro-inflammatory factors released during sepsis and pathogenic exosomes derived from M1 macrophages, the development of biomimetic nanosponges (such as macrophage membrane-coated nanoparticles) or specific antibodies for circulatory interception and neutralization can effectively block the cascade diffusion of inflammatory signals to pulmonary vascular endothelial and epithelial cells (84). This therapeutic strategy not only avoids the risk of secondary infection caused by systemic immunosuppression but also fundamentally reshapes the immune ecology of the lung microenvironment.

## 6 Endothelial cell-derived exosomes: vascular leakage, immunothrombosis, and cuproptosis

Injury to pulmonary microvascular endothelial cells is the fundamental cause of the exudation of protein-rich edema fluid in sepsis-associated ARDS. In this pathological process, the role of endothelial-derived exosomes has far exceeded that of simple byproducts of injury; they are both molecular messengers leading to changes in vascular permeability and catalytic surfaces mediating immunothrombosis.

### 6.1 Dysregulation of the Angiopoietin-Tie2 axis and vascular barrier disintegration

The maintenance of vascular endothelial permeability is highly dependent on the binding of Angiopoietin-1 (Ang-1) to the Tie2 receptor on the surface of endothelial cells, a homeostasis that is a core mechanism guaranteeing vascular maturation and stability (85, 86). However, sepsis disrupts this balance, and endothelial-derived exosomes (Endo-Exos) play a key negative regulatory role therein.

Under sepsis stress, activated endothelial cells secrete Ang-2 via the exosomal pathway. These Ang-2-rich exosomes or the proteins they carry act as antagonistic ligands for the Tie2 receptor, acting on neighboring endothelial cells to competitively inhibit Tie2 receptor activation (87, 88), thereby disrupting vascular homeostasis and promoting inflammatory responses. More critically, this process induces the phosphorylation, internalization, and lysosomal degradation of Vascular Endothelial Cadherin (VE-Cadherin), the core protein of adherens junctions, thereby unzipping the tight junctions between cells (89).

Furthermore, Endo-Exos exert a dual destructive effect in endothelial injury: they not only serve as carriers to synergistically transport matrix metalloproteinases (such as MMP-2 and MMP-9), directly enzymatically degrading vascular basement membrane components (90), but also target and inhibit mTOR kinase via the pathogenic miR-99a/b they are enriched with, driving sustained inflammatory responses during sepsis (91). Conversely, endogenous defense mechanisms for endothelial homeostasis are also severely hit. The protective molecule miR-126-3p shows characteristic depletion in sepsis plasma and microvesicles (92); this loss relieves the inhibition of target genes, leading to uncontrolled expression of adhesion molecules like VCAM-1, marking the total collapse of the endothelial defense system (93). On this basis, molecules such as miR-125a-5p and miR-200c also synergistically intervene in the pathological process, further exacerbating permeability disorders at the molecular level by disrupting cytoskeletal remodeling and regulating VE-cadherin transcription factors (94, 95). This multidimensional molecular strike ultimately triggers the physical disintegration of the microvascular barrier, allowing protein-rich fluid to seep unimpeded into the alveolar space, forming the characteristic non-cardiogenic pulmonary edema of ARDS.

### 6.2 The reciprocal loop of immunothrombosis and NETosis

Sepsis is often accompanied by widespread microvascular thrombosis, leading to organ dysfunction. Existing research indicates that the formation of immunothrombosis involves complex intercellular crosstalk, including the extensive participation of exosomes released by endothelial cells and neutrophil extracellular traps (NETs), which jointly promote pathological thrombo-inflammatory responses (17).

At the molecular level, endothelial cell-derived exosomes initiate the coagulation process through membrane surface remodeling. Damaged endothelial cells and the microparticles they release undergo a loss of membrane lipid asymmetry, leading to the externalization and exposure of negatively charged phosphatidylserine (PS) (96). This high-affinity catalytic plane provides necessary sites for the anchoring and assembly of prothrombinase complexes (Tenase and Prothrombinase), thereby exponentially amplifying the efficiency of thrombin generation (97).

At the cellular level, the interaction between endothelial cells and neutrophils constitutes the core hub of immunothrombosis. Damaged endothelium not only stimulates neutrophils to burst-release NETs, but NETs in turn induce endothelial cells to highly express biologically active tissue factor (TF), forming a malignant pro-coagulant positive feedback loop (17). Subsequently, the highly viscous DNA scaffold structure of NETs serves as a platform to further capture circulating activated platelets and enrich tissue factor (TF), jointly constructing stable immunothrombi, thereby exacerbating the pathological process (16). This process widely obstructs pulmonary capillaries, significantly aggravating microcirculatory dysfunction and hypoxia.

### 6.3 Cuproptosis: a new metabolic horizon of vascular injury

Cuproptosis, as a novel form of regulated cell death distinct from oxidative stress-driven ferroptosis, is essentially mitochondrial metabolic toxicity triggered by intracellular copper ion overload (98). In the highly inflammatory background of sepsis, serum copper levels often rise significantly as acute-phase reactants; this systemic imbalance in copper homeostasis constitutes the initiating factor for pulmonary microvascular endothelial cell injury (99). Unlike ferroptosis which primarily attacks membrane lipids, cuproptosis specifically targets the tricarboxylic acid (TCA) cycle machinery of cells. Excess copper ions directly bind to and target lipoylated components in the mitochondrial TCA cycle, particularly dihydrolipoamide S-acetyltransferase (DLAT), leading to the toxic aggregation of related mitochondrial proteins and the instability of iron-sulfur cluster proteins (98). This process triggers severe proteotoxic stress and blocks the cellular energy metabolism hub, ultimately leading to irreversible mitochondria-dependent death in metabolically active pulmonary vascular endothelial cells.

In this pathological process, it is theoretically plausible that the functional dimensions of endothelial-derived exosomes extend beyond being passive carriers of metabolic waste. While existing research has confirmed that dysregulated expression of copper transporters (such as ATP7A, CTR1) is a core mechanism inducing cellular cuproptosis in models of endothelial injury induced by disturbed blood flow (100, 101), their exact relationship with exosomal transport in sepsis remains largely unexplored.

Drawing upon the known property of exosomes to targetedly deliver bioactive molecules, we raise a speculative hypothesis: exosomes released by damaged endothelium might have the theoretical capacity to induce secondary cuproptosis responses in the vascular microenvironment, potentially by encapsulating abnormal copper transporters or cytotoxic lipoylated protein aggregates. Furthermore, from a purely conceptual standpoint, one might postulate that metabolic reprogramming signals carried by these exosomes could disrupt the metabolic plasticity of recipient cells, blocking their adaptive transition from oxidative phosphorylation to glycolysis and thereby imprisoning cells in a metabolic phenotype highly sensitive to cuproptosis (99, 102).

**Figure 3:**
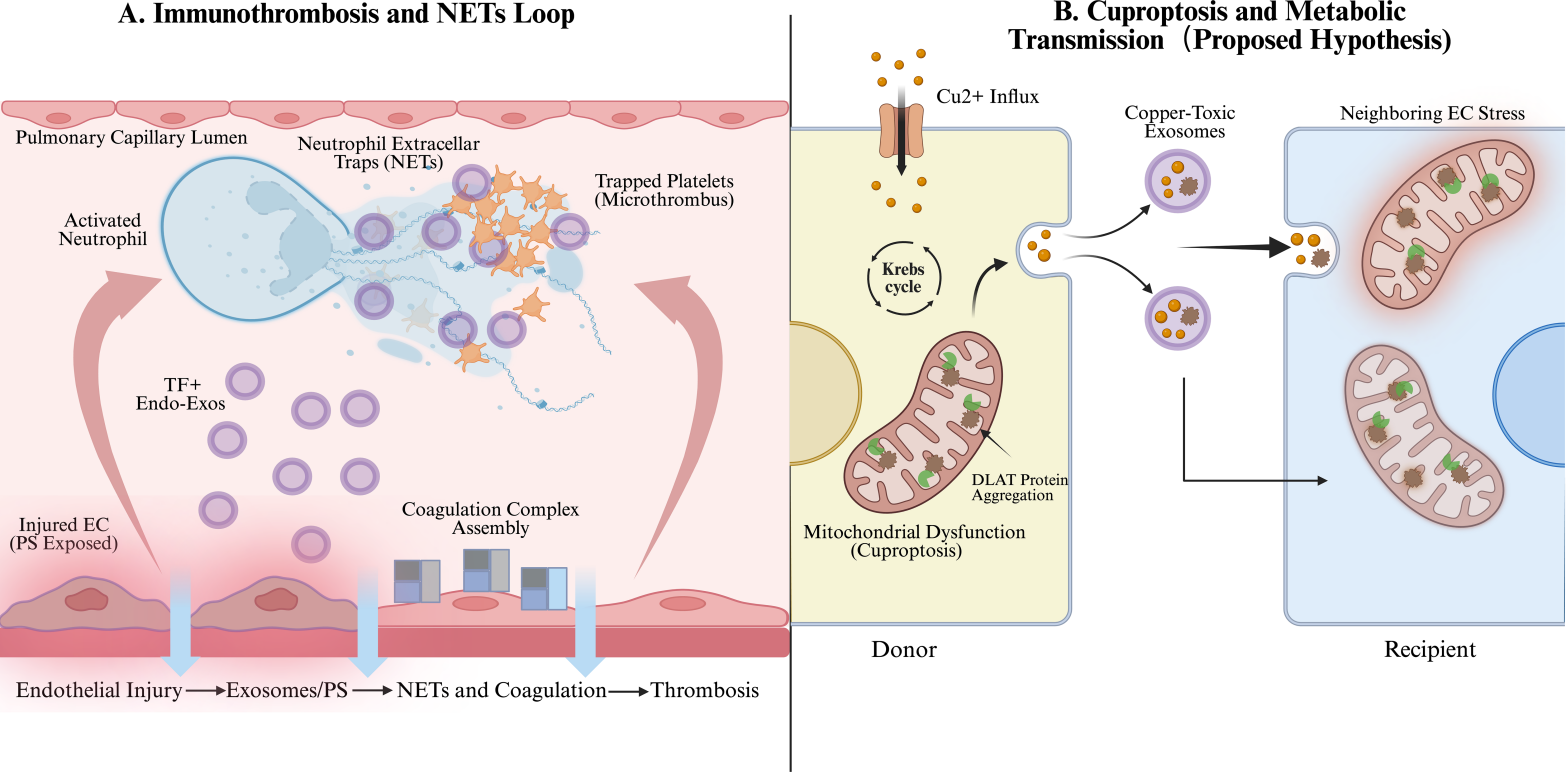
Dual mechanisms of vascular endothelial dysfunction in sepsis-associated ARDS. Immunothrombosis and Cuproptosis propagation. This figure illustrates two key pathological dimensions driven by endothelial-derived exosomes in pulmonary microcirculatory dysfunction. **(A)** Immunothrombosis and NETs Loop (Left): Under sepsis stress, the membrane phosphatidylserine (PS) of damaged endothelial cells is externalized (red halo), and exosomes rich in tissue factor (TF) are released (purple spheres) (96). These exosomes not only serve as catalytic platforms for coagulation complex assembly but also synergize with neutrophil extracellular traps (NETs) to capture platelets and form microthrombi, constructing a malignant positive feedback loop of thrombo-inflammation (16, 17). **(B)** Cuproptosis and Metabolic Transmission (Right): Intracellular copper ion overload leads to toxic aggregation of mitochondrial DLAT proteins (brown clumps) in donor cells, inducing cuproptosis (98). Damaged cells release “copper-toxic exosomes” carrying these aggregates, which, upon uptake by neighboring recipient cells, cascade mitochondrial dysfunction and metabolic rigidity within the vascular bed, leading to diffuse vascular injury (100, 101).

We must strongly emphasize that this exosome-mediated cuproptosis propagation network is currently entirely conceptual and lacks direct empirical evidence in ARDS models. If such a paracrine mechanism is experimentally validated in the future, it could provide a novel explanation for why focal endothelial injury rapidly expands into diffuse pulmonary microvascular barrier collapse. Until then, rigorous *in vivo* and in intro experimental studies are urgently required to determine whether specific sorting of copper-related cargoes into endothelial exosomes actually occurs, and to delineate their exact roles in recipient cells. Nevertheless, based on this theoretical framework, the targeted blockade of copper transport or the use of copper chelators remains a promising avenue for future investigation into protecting the integrity of the septic pulmonary vasculature (10) (**Figure 3****).**

### 6.4 Translational medicine value: real-time monitoring window for microvascular leakage and thrombosis risk

Unlike alveolar epithelial cell-derived exosomes which usually require invasive bronchoalveolar lavage for acquisition, pulmonary vascular endothelial cells directly face the blood circulation, allowing the endothelial-derived exosomes they release to enter peripheral blood unimpeded (103). This unique anatomical advantage makes them ideal liquid biopsy targets for monitoring the pathological state of the pulmonary microcirculation (104). Increasing evidence suggests that fluctuations in the concentration of circulating endothelial exosomes and their phenotypic evolution can reflect the risk of vascular leakage and embolism in sepsis-associated ARDS earlier and more sensitively than traditional clinical indicators.

#### 6.4.1 Molecular quantification of barrier disintegration and correlation with lung injury severity

The membrane surface of endothelial exosomes retains characteristic antigens of parent cells, providing clinicians with a molecular code to interpret specific mechanisms of vascular barrier injury. The levels of CD144 (VE-cadherin) or CD31-positive exosomes in circulation directly reflect the physical dissociation of endothelial intercellular junction complexes (105), while the accompanying high expression of Ang-2 further confirms the loss of shear stress sensing mechanisms and severe microvascular injury (106). Clinical correlation analyses confirm that the levels of these endothelial injury markers are significantly correlated with the severity of lung injury (e.g., decline in oxygenation index) (107). This means that before diffuse infiltration appears on chest imaging, the degree of pulmonary microvascular leakage can be quantitatively assessed by detecting these molecular markers, thereby capturing the real-time window of microvascular permeability changes.

#### 6.4.2 Early warning and risk stratification of immunothrombosis

Besides reflecting physical barrier destruction, endothelial-derived exosomes are key indicators for assessing the risk of microvascular immunothrombosis formation. Sepsis-induced endothelial-derived exosomes are often accompanied by high expression of tissue factor and adhesion of citrullinated histone H3, molecular features that reveal the malignant interaction between endothelial injury and neutrophil extracellular traps (NETs) (105, 108). Clinical studies further confirm that the levels of endothelial-derived exosomes and vesicles in the plasma of septic shock patients are significantly associated with the occurrence of early disseminated intravascular coagulation (DIC), serving as potential biomarkers for assessing vascular injury (109). Based on this, by combined monitoring of exosome subsets reflecting leakage and pro-coagulation, clinicians hope to construct an ARDS subtyping model based on pathological mechanisms, thereby identifying high-risk patients requiring intensified vascular protection or anticoagulant therapy, and moving the intervention window forward from organ failure to the early stage of vascular injury.

## 7 Other cell-derived exosomes: synergistic pathogenicity and repair mechanisms in multidimensional networks

In the complex pathological microenvironment of sepsis-associated ARDS, in addition to the core axis composed of alveolar epithelium, endothelium, and macrophages, exosomes derived from neutrophils, platelets, and mesenchymal stem cells play equally crucial roles. These nanovesicles are not only molecular amplifiers of the cytokine storm and coagulation disorders but also key regulators initiating endogenous repair, collectively weaving a dynamic communication network that determines the fate of lung tissue.

### 7.1 Neutrophil-derived exosomes: molecular engines driving the NETosis cascade and microcirculatory dysfunction

As the vanguard of the innate immune system, the role of neutrophils in sepsis-associated ARDS has transcended simple pathogen clearance; their excessive activation and the release of neutrophil extracellular traps (NETs) constitute the core mechanism of immunopathological injury (110). Recent research perspectives reveal that neutrophil-derived exosomes are not simple metabolic byproducts but key signal carriers driving the cascade amplification of inflammation and sustaining the high-inflammatory state of the lung.

These exosomes construct a feed-forward loop of inflammatory amplification within the microenvironment. They act on resting neutrophils via paracrine mechanisms and activate inflammatory signaling pathways such as p38 MAPK, thereby inducing secondary NETosis (111). These exosomes highly express integrin molecules such as Mac-1 on their surface, endowing them with the ability to adhere to fibrinogen and the vascular endothelial surface and providing a physical scaffold for NET anchoring, leading to an exponential amplification of the local pulmonary inflammatory response (112).

Beyond acting as inflammatory amplifiers, neutrophil exosomes are hidden drivers of pulmonary microvascular barrier disruption, with their pathogenic mechanisms presenting a dual strike feature of structural destruction and repair inhibition. Structurally, these exosomes are rich in high concentrations of myeloperoxidase (MPO) and serine proteases (such as Cathepsin G), which can directly enzymatically degrade tight junction proteins between endothelial cells and the glycocalyx on the vascular surface, leading to physical barrier disintegration (113). Simultaneously, when these vesicles are endocytosed by endothelial cells, the miR-142-3p they release can target and inhibit key endothelial repair signaling pathways, thereby hindering the regeneration and integrity restoration of damaged blood vessels at the post-transcriptional level (114). This pathological mechanism manifests as significant biomarker correlation clinically; studies confirm that the levels of neutrophil-derived Cluster of Differentiation 15 (CD15)-positive exosomes in plasma are significantly positively correlated with patients’ APACHE II scores, directly reflecting disease severity (115). Based on this, further combined detection of circulating markers such as Citrullinated Histone H3 (CitH3) or MPO-DNA complexes has been proven effective in quantifying systemic and pulmonary inflammatory burden and microthrombotic status, thus providing an indispensable non-invasive window for assessing intrapulmonary NETosis intensity and precisely screening populations that would benefit from anti-NETs therapy (116).

### 7.2 Platelet-derived exosomes: the microcirculatory hub driving thrombo-inflammation

In the complex pathophysiological network of sepsis-associated ARDS, platelets have transcended traditional hemostatic functions, acting as key immune effector cells in the circulatory system. The exosomes released upon their activation (P-Exos), by virtue of their nanoscale size and high biological stability, build a molecular bridge connecting endothelial injury and immune activation within the pulmonary microcirculation, serving as core mediators driving the formation of the thrombo-inflammatory microenvironment.

First, P-Exos act as highly mobile pro-coagulant catalytic platforms, initiating the coagulation cascade through pathological membrane lipid remodeling. The externalization and exposure of phosphatidylserine (PS) on the P-Exos membrane surface provide a high-density negatively charged plane, greatly promoting the assembly of the prothrombinase complex; its catalytic efficiency per unit surface area can reach 50 to 100 times that of activated platelets themselves (117). More critically, P-Exos break the spatial limitations of coagulation reactions on the vessel wall. Mechanistic studies indicate that exosomes carrying tissue factor (TF) can fuse with platelets (118), while protein disulfide isomerase (PDI) plays a key molecular switch role in activating tissue factor on the surface of these exosomes (119). This mechanism endows P-Exos with the ability to deliver active coagulation factors deep into the pulmonary capillary bed, thereby driving widespread microthrombosis and ventilation/perfusion mismatch.

In addition to directly driving coagulation, P-Exos are key relayers maintaining and amplifying the pulmonary cytokine storm. Under sepsis conditions, activated platelets and their released P-Exos specifically enrich damage-associated molecular patterns (DAMPs) such as high mobility group box 1 (HMGB1). Upon uptake by neutrophils, they can strongly induce the explosive release of NETs (120). This process constructs a malignant positive feedback loop: released NETs act as thrombotic scaffolds to further capture circulating platelets and induce their activation, promoting further P-Exos release, and ultimately leading to widespread occlusion of pulmonary microvessels. At the clinical translation level, P-Exos demonstrate important liquid biopsy value; stable binding of CD62P (P-selectin) on their membrane surface can truly reflect the sustained activation burden of platelets *in vivo* (121), while mitochondrial DNA (mtDNA) enriched in their contents has been confirmed as a key danger molecule driving systemic inflammatory responses and multiple organ failure (122).

### 7.3 Mesenchymal stem cell-derived exosomes: bioenergetic resuscitation and immune microenvironment remodeling

Unlike the injury cascades driven by the aforementioned pathogenic exosomes, mesenchymal stem cell-derived exosomes (MSC-Exos) represent a key counter-regulatory mechanism by which the organism resists inflammatory strikes and initiates regeneration. These nanovesicles provide multidimensional therapeutic support for the pathological outcome of sepsis-associated ARDS through unique organelle transfer and immunometabolic reprogramming.

One of the core repair mechanisms of MSC-Exos lies in directly driving the bioenergetic resuscitation of damaged effector cells. Studies confirm that MSCs can transfer healthy mitochondria to damaged alveolar macrophages via extracellular vesicles or direct intercellular contact (such as tunneling nanotubes) (123). This transfer can enhance oxidative phosphorylation levels in recipient cells and restore ATP production, effectively reversing endotoxin-induced cellular energy metabolic dysfunction (124), thereby significantly reducing pulmonary microvascular permeability and promoting pulmonary edema resolution.

Furthermore, this class of exosomes promotes tissue repair by remodeling the immune microenvironment. For example, MSC-Exos carrying let-7b and miR-21-5p have been confirmed to precisely inhibit the TLR4/NF-κB signaling pathway, reversing the pro-inflammatory phenotype of macrophages (125, 126). This anti-inflammatory polarization effect not only aids in the healing of chronic wounds or ischemic tissues but also provides a key immunological basis for alveolar regeneration and barrier repair following lung injury. Synergistically, at the vascular repair level, Ang-1 mRNA delivered by MSC-Exos can induce damaged endothelium to express stabilizing factors, directly antagonizing vascular leakage (127). At the clinical translation level, in addition to using classic surface markers such as CD90 and CD73 to establish the identity of MSC-Exos (128), clinical trials further indicate that a reduction in plasma Angiopoietin-2 (Ang-2) levels suggests improvement in endothelial injury and is closely related to the biological effects of MSC therapy (129), allowing it to be constructed as a precise molecular ruler for assessing tissue repair potential.

In summary, the pathological mechanisms of sepsis-associated ARDS are not limited to injury of a single cell type but involve complex intercellular network communication mediated by multi-source exosomes. These exosomes not only transmit signals between similar cells but also achieve cross-barrier bidirectional regulation (Crosstalk) between alveolar epithelium and vascular endothelium. For instance, injury signals released by epithelial cells can lead to endothelial barrier dysfunction via exosomes, while endothelial-derived exosomes may conversely affect epithelial metabolism and repair. This cross-cell type cascade amplification effect and positive feedback loop may be the key microenvironmental basis for the sustained progression of sepsis-induced lung injury. To systematically organize this complex network, we summarize the core cargoes, targeting mechanisms, and biological effects of exosomes from different cell sources in (**Table 1**), aiming to provide a theoretical basis for blocking this cascade reaction chain.

**Table 1:** Summary of key cargoes, targeting mechanisms, and biological effects of cell-specific exosomes in sepsis-associated ARDS.

Source cell	Key exosome cargo	Recipient cell	Mechanism/pathway	Biological effect	Ref.
Alveolar Epithelial Cell	miR-92a-3p	Alveolar Macrophage	Post-transcriptional regulation	Promotes activation; Amplifies inflammation	(12)
	lncRNA Rmrp/STIMATE	Alveolar Macrophage	Immunometabolic reprogramming/Calcium signaling	Induces immunosuppression/Regulates macrophage polarization	(39, 40)
	Tenascin-C	Macrophage	Activates TLR4 pathway	Induces pyroptosis, exacerbates cytokine storm	(41)
	GSDMD, Caspase-1	Neighboring epithelial/immune cells	Pyroptosis signal cascade	Propagates pyroptosis; Disintegrates barrier	(42, 43)
	Oxidized lipids (4-HNE); GPX4 (Depleted)	Alveolar Epithelial Cell	Transmits oxidative stress; Depletes antioxidants	Propagates ferroptosis in a wave-like manner	(46, 47)
	Oxidized mtDNA (Mito-Exosomes)	Alveolar Macrophage	cGAS-STING/TLR9	Activates innate immunity, induces neutrophil recruitment	(51, 52)
Alveolar Macrophage	PKM2, HK2 (M1 type)	Neighboring cells/Autocrine	HIF-1α/Glycolytic reprogramming	Maintains Warburg effect (High inflammation)	(27, 72)
	miR-155 (M1 type)	Multiple cell types	Targets and inhibits SOCS1	Releases inflammation brakes, promotes NF-κB activation	(13–15)
	Lactylated histone (H3K18la); HMGB1	Multiple cell types	Epigenetic modification	Solidifies inflammatory transcription, reflects metabolic-epigenetic crosstalk	(11, 76, 77)
	Succinylated protein (PKM2)	Neighboring cells/Autocrine	Activates NLRP3/AIM2	Exacerbates pyroptosis and IL-1β release	(79, 80)
	miR-223 (M2 type)	Multiple cell types	Inhibits NLRP3/STAT3	Anti-inflammatory effect, attenuates tissue injury	(73, 74)
	BMPR2 (M2 type)	Type II Alveolar Epithelial Cell	BMPR1B-SMAD1-ID1	Promotes epithelial proliferation and transdifferentiation (Tissue regeneration)	(75)
Endothelial Cell	Angiopoietin-2 (Ang-2)	Endothelial Cell	Antagonizes Tie2 receptor	Disrupts homeostasis; Increases permeability	(87, 88)
	MMP-2, MMP-9	Vascular Basement Membrane	Enzymatically degrades basement membrane	Physically destroys microvascular barrier	(90)
	miR-99a/b	Endothelial Cell	Inhibits mTOR	Drives sustained inflammatory response	(91)
	Copper transporters/Complexes (Hypothesis)	Vascular Endothelial Cell	Interferes with copper homeostasis/Mitochondrial metabolism	Induces cuproptosis cascade	(10, 100–102)
Neutrophil	MPO, Cathepsin G	Endothelial Cell	Degrades junction proteins/Glycocalyx	Destroys vascular endothelial integrity	(113)
	miR-142-3p	Endothelial Cell	Targets and inhibits repair pathways	Hinders regeneration of damaged vessels	(114)
	Integrin Mac-1	Fibrinogen/Endothelial Surface	Physical adhesion/Scaffolding	Promotes NETs anchoring and inflammatory amplification	(112)
Platelet	Tissue Factor (TF), PDI	Coagulation System	Exposes PS/Activates TF	Initiates coagulation cascade, forms immunothrombosis	(117–119)
	HMGB1	Neutrophil	Induces autophagy	Triggers explosive release of NETs	(120)
Mesenchymal Stem Cell	Functional Mitochondria	Alveolar Macrophage	Mitochondrial transfer	Restores oxidative phosphorylation, reverses energy metabolic dysfunction	(123, 124)
	let-7b, miR-21-5p	Macrophage	Inhibits TLR4/NF-κB	Induces M2-type anti-inflammatory polarization	(125, 126)
	Ang-1 mRNA	Endothelial Cell	Promotes Ang-1 expression	Antagonizes vascular leakage, stabilizes barrier	(127)

## 8 Toward clinical translation: from single biomarkers to multidimensional precision medicine systems

Although cell-specific exosomes have demonstrated immense potential in revealing the pathological mechanisms of sepsis-associated ARDS, translating them from the laboratory into tools that guide clinical decision-making still requires the construction of a standardized system integrating diagnosis, subtyping, and treatment monitoring. Future clinical applications will no longer be limited to the detection of single indicators but will evolve towards multi-parametric, automated, and visualized directions.

### 8.1 Constructing panoramic injury atlases and molecular subtyping strategies

Faced with the high biological heterogeneity of sepsis-associated ARDS, relying solely on exosomes from a single cell source is insufficient to capture the full picture of the disease. Building a dual-dimensional assessment model covering both structural injury and functional dysfunction is key to breaking through existing diagnostic bottlenecks.

First, a combinatorial detection strategy specific to cell sources should be implemented. By jointly analyzing markers representing physical disruption of the alveolar epithelial barrier (such as RAGE-positive exosomes) and markers reflecting high permeability of the vascular endothelium (such as CD31 or Ang-2-positive exosomes), clinicians can capture barrier injury at the microscopic level during the latent period before radiographic changes appear. This integrated strategy can precisely distinguish target organ injury from non-specific systemic inflammatory responses, significantly enhancing diagnostic specificity.

Second, a dynamic ratio model based on exosomal cargo aids in achieving precise immune subtyping. Research indicates that ARDS patients exist in two distinct molecular phenotypes: hyper-inflammatory and hypo-inflammatory, with significant differences in therapeutic response (130). By calculating the ratio of pro-inflammatory exosomes (such as macrophage-derived miR-155-positive exosomes) to reparative exosomes (such as MSC-derived Ang-1 or endothelial cell-derived miR-126-positive exosomes), a quantitative indicator reflecting the organism’s immune balance state can be constructed. This indicator can not only warn of the occurrence of a cytokine storm earlier than traditional inflammatory factors but also identify patient subgroups in the immune paralysis stage, thereby providing an objective basis for the precise application of immunomodulatory drugs such as glucocorticoids.

### 8.2 Next-generation detection technologies: microfluidic chips and surface-enhanced raman scattering

Traditional ultracentrifugation methods, being time-consuming and dependent on large equipment, cannot meet the demand for bedside point-of-care testing (POCT) for critically ill patients in the ICU. Advances in micro- and nanofabrication technologies are driving exosome detection toward automation and miniaturization, providing technical support to solve the timeliness problem (131).

Microfluidic chips based on acoustofluidic or magnetic bead sorting technologies also demonstrate huge application prospects. For example, new-generation acoustofluidic platforms can complete the high-efficiency, label-free isolation and enrichment of exosomes from microliter-volume blood samples within minutes (132). This technology not only drastically shortens sample processing time but also reduces human operation error through integrated design. Furthermore, techniques combining specific probes hold promise for the direct capture and analysis of exosome subsets carrying lung-specific antigens (such as KL-6, SP-B) (133).

To further enhance detection sensitivity and multiplexing capabilities, Surface-Enhanced Raman Scattering (SERS) technology is being introduced into the field of exosome analysis. Combined with specific SERS nanotags, this technology can achieve high-sensitivity quantitative analysis of multiple protein markers on the exosome surface (134). Additionally, by mapping the Raman spectral fingerprints of exosomes and combining them with machine learning algorithms, clinicians may hope to simultaneously assess the degree of epithelial injury, immune activation status, and coagulation disorder risk in a single test, truly realizing a panoramic decoding of the pathophysiological state of ARDS (135).

### 8.3 New horizons in therapeutic applications: nebulization and organelle transplantation

In the therapeutic dimension, mesenchymal stem cell (MSC)-derived exosomes, as a cell-free therapy, are undergoing dual innovations in administration routes and mechanisms of action. Optimization of the administration route is key to improving efficacy. Traditional intravenous injection often leads to massive sequestration of exosomes by the mononuclear phagocyte system of the liver and spleen, greatly limiting their bioavailability in the lung. Recent preclinical studies and early clinical trials tend to adopt nebulization inhalation strategies (136). Nebulization allows exosomes to directly reach the alveolar surface and bronchioles, cross the mucus layer, and be specifically taken up by damaged epithelial cells, significantly increasing local drug concentration while avoiding the risk of metabolic inactivation associated with systemic administration (65).

At the mechanistic level, the therapeutic potential of MSC exosomes has transcended simple molecular delivery, extending into the field of organelle transplantation. Research confirms that MSC exosomes can directly transfer functional mitochondria to key effector cells such as damaged macrophages, thereby rapidly restoring ATP generation and improving bioenergetic metabolism (119). Furthermore, these exosomes can effectively reverse ferroptosis and cell exhaustion induced by metabolic failure by activating antioxidant signaling pathways (such as the SIRT1/NRF2 axis), providing a novel therapeutic dimension for the metabolic resuscitation of severe ARDS (137).

### 8.4 Standardization challenges and implementation of MISEV guidelines

Despite promising prospects, the clinical translation of exosomal markers still needs to overcome the dual barriers of technical feasibility and standardization. The greatest challenge currently faced is the trade-off between cell specificity and detection timeliness. Although tracing strategies based on membrane proteins like RAGE or CD31 are theoretically feasible, in the real-world scenario of ICU emergency care, existing immunoaffinity technologies often take too long and are too costly to rapidly enrich rare exosome subsets of specific origins (such as alveolar epithelial origin) with high purity from complex plasma components (138). In this regard, the MISEV guidelines released by the International Society for Extracellular Vesicles emphasize that while pursuing marker specificity, separation purity and quantitative accuracy must be strictly evaluated; this is the prerequisite for ensuring data reproducibility (6).

Furthermore, as underscored by the MISEV guidelines, clinical translation is strongly contingent upon pre-analytical variables, particularly the choice of sample source. Different biological fluids present distinct applicable scenarios, advantages, and inherent biases. Bronchoalveolar lavage fluid (BALF) offers the most direct anatomical window into the alveolar microenvironment, making it the optimal source for capturing alveolar epithelial-derived exosomes (such as RAGE-positive vesicles) and assessing local immune responses. However, its invasive nature severely limits repeated sampling in hemodynamically unstable or severely hypoxemic ARDS patients. Additionally, the unpredictable dilution effect inherent to the lavage procedure introduces significant challenges for absolute exosome quantification (139). Conversely, peripheral blood provides a non-invasive avenue for real-time dynamic monitoring, particularly suitable for tracking endothelial-derived and systemic immune cell-derived exosomes. Among blood fractions, plasma is highly preferred over serum for exosome analysis. The coagulation cascade required for serum preparation triggers massive *in vitro* platelet activation, artificially releasing abundant platelet-derived exosomes that severely skew and mask the true *in vivo* circulating exosomal profile (140, 141). Therefore, platelet-depleted plasma, prepared via standardized centrifugation protocols, remains the recommended matrix for systemic liquid biopsy in ARDS.

Even when utilizing plasma, sepsis complicated by multiple organ dysfunction leads to a large number of extra-pulmonary exosomes mixing into the circulation, severely interfering with the precise tracing of lung injury. The key to breaking this bottleneck lies in constructing a dual-positive detection strategy. Recent research confirms that pulmonary microvascular endothelial cells specifically highly express Angiotensin-Converting Enzyme (ACE) (142); combined detection of a universal endothelial antigen and ACE can effectively filter out background noise from the systemic circulation, thereby precisely assessing pulmonary microvascular injury and predicting the risk of ARDS occurrence.

Additionally, high-abundance lipoproteins in clinical samples are major interfering factors; they are similar in size and density to exosomes, often leading to false-positive results (143). Therefore, future clinical detection workflows need to develop towards automation, developing microfluidic systems integrating Size Exclusion Chromatography (SEC) with immunoaffinity to maximize the balance between decontamination efficiency and detection speed (144). Simultaneously, data normalization is another major challenge. It is recommended to introduce external synthetic spike-in controls or use biologically significant ratio indicators in the detection system to eliminate systematic errors, thereby ensuring the robustness and cross-center comparability of diagnostic results (145).

## 9 Conclusion and outlook

The reason sepsis-associated ARDS has long faced the clinical dilemma of high mortality is largely attributed to the current diagnostic system’s lack of resolution regarding pathological mechanisms. Research on cell-specific exosomes provides a novel perspective for understanding this complex syndrome; they are not only messengers of intercellular communication but also executors driving ferroptosis propagation, immunometabolic reprogramming, and organ crosstalk injury.

Facing the challenge of clinical heterogeneity brought by the 2023 Global New Definition of ARDS, exosome-based liquid biopsy offers a molecular phenotyping solution. By integrating epithelial- and endothelial-specific exosomes, we hope to capture microscopic injury before radiographic changes appear, achieving ultra-early warning. By analyzing the metabolic and functional cargoes of macrophage and neutrophil exosomes, we can distinguish patients with hyper-inflammatory, hyper-thrombotic, or hyper-suppressive phenotypes, guiding the precise implementation of anti-inflammatory, anticoagulant, or immunomodulatory treatments. Furthermore, by dynamically tracking MSC exosome markers and the recovery of mitochondrial function, we can assess therapeutic responses and tissue repair progress in real-time.

Future research should focus on conducting large-scale, multi-center prospective cohort studies to validate the clinical efficacy of the aforementioned markers. Integrating clinical phenotypic data with exosomal multi-omics data using artificial intelligence algorithms to construct an “exosome-clinical” multimodal prediction model will be the key path to propelling ARDS diagnosis and treatment from “syndrome management” to “mechanism-driven precision medicine,” ultimately bringing about a substantial breakthrough in reducing the high mortality rate of sepsis patients.
